# High-protein enteral nutrition in intensive care unit patients undergoing advanced early mobilization: protocol for a randomized controlled trial

**DOI:** 10.1186/s13063-026-09584-9

**Published:** 2026-02-21

**Authors:** Ginga Suzuki, Yuji Iwanami, Masayoshi Nakazawa, Shunta Yoshioka, Masashi Furuta, Masaru Nakanishi, Hiromi Kanayama, Kohei Ishikawa, Saria Nishioka, Toshimitsu Kobori, Yuka Masuyama, Saki Yamamoto, Yui Shimanuki, Hibiki Serizawa, Yoshimi Nakamichi, Mitsuru Honda, Naohiro Washizawa, Ikuko Okuni

**Affiliations:** 1 Critical Care Center, Toho University Omori Medical Center, 6-11-1, Omori Nish, Ota-ku, Tokyo Japan; 2 Department of Rehabilitation Medicine, Toho University School of Medicine, 6-11-1, Omori Nishi, Ota-ku, Tokyo Japan; 3 Department of Nutrition Management, Toho University Omori Medical Center, 6-11-1, Omori Nishi, Ota-ku, Tokyo Japan

**Keywords:** Intensive care unit, Randomized controlled trial, Protein intake, Early mobilization

## Abstract

**Background:**

In critically ill patients admitted to the intensive care unit (ICU), early nutritional therapy and mobilization rehabilitation are considered important interventions; however, when applied independently, neither approach has consistently demonstrated clear benefits in improving clinical outcomes. To date, no study has specifically examined the potential synergistic effects of combining high-protein nutrition with early mobilization. Therefore, this trial aims to investigate whether combining these interventions can promote physical and functional recovery after ICU discharge.

**Methods:**

This single-center, prospective, participant- and assessor-blinded randomized controlled trial with a planned sample size of 90 critically ill patients will be conducted in the ICU of a tertiary emergency care facility. Mechanically ventilated adult patients in the ICU will be eligible. Participants will be randomly assigned to either a “usual-protein group” (1.2 g/kg/day) or a “high-protein group” (1.8 g/kg/day). In both groups, early mobilization will be delivered according to a standardized protocol using a mobile patient lift, enabling patients to achieve standing or higher-level activities early in the ICU course. The primary outcome will be the motor score of the Functional Independence Measure (FIM) at hospital discharge. Secondary outcomes will include the Cognitive subscale of the Functional Independence Measure, Brief Pain Inventory score, ICU length of stay, hospital length of stay, and discharge disposition. Written informed consent will be obtained from participants or their legally authorized representatives prior to enrollment.

**Discussion:**

This trial will be the first to evaluate the potential synergistic effects of high-protein nutrition and standardized early high-intensity mobilization in ICU patients. By focusing on protein dose as the sole intervention variable and using a mobile patient lift to ensure uniform mobilization intensity, the study is designed to provide robust evidence regarding the role of high-protein nutrition during intensive rehabilitation. This trial is conducted in accordance with the SPIRIT guidelines. The findings are expected to inform future multicenter trials and contribute to the development of clinically relevant strategies to improve post-ICU functional recovery.

**Trial registration:**

University Hospital Medical Information Network Clinical Trials Registry (UMIN-CTR), UMIN000059233. Registered on September 29, 2025. https://center6.umin.ac.jp/cgi-open-bin/ctr_e/ctr_his_list.cgi?recptno=R000067751.

**Supplementary Information:**

The online version contains supplementary material available at 10.1186/s13063-026-09584-9.

## Background

In the intensive care unit (ICU), comprehensive care that addresses not only acute management but also long-term functional outcomes has become increasingly important [1–3]. Among the strategies proposed, adequate protein delivery and early mobilization are widely recognized as key interventions aimed at mitigating muscle loss [4, 5] and preventing the development of post-intensive care syndrome [6–8]. International guidelines recommend protein intake of 1.2–2.0 g/kg/day to promote muscle protein synthesis and improve functional outcomes [9, 10].

Malnutrition is highly prevalent among critically ill patients. A systematic review that used validated nutritional assessment tools reported that 38–78% of patients were malnourished at or shortly after ICU admission [11]. Importantly, malnutrition diagnosed by formal nutritional assessment was independently associated with adverse clinical outcomes, including increased ICU length of stay, ICU readmission, infectious complications, and hospital mortality, even after adjustment for illness severity and comorbidities. These findings underscore malnutrition as a clinically meaningful and independent risk factor for poor outcomes in the ICU and highlight the urgency of optimizing nutritional strategies in this population.

However, accumulating evidence indicates that higher protein intake does not uniformly translate into better clinical outcomes. Recent randomized controlled trials (RCTs) have reported that protein delivery exceeding 1.2 g/kg/day, when administered as a stand-alone intervention, does not significantly improve patient-centered outcomes [12] and may even be harmful in certain clinical contexts [5, 13]. Observational studies further suggest that the relationship between protein intake and outcomes such as mortality may follow a U-shaped pattern, with moderate protein targets being associated with more favorable short-term outcomes, while higher targets confer no additional benefit [14, 15]. Consistent with these findings, an updated meta-analysis has reported heterogeneous effects of higher protein intake on clinical outcomes, highlighting the limitations of protein supplementation as a stand-alone intervention [5]. Similarly, although various early mobilization strategies, including in-bed exercise and out-of-bed activities, have been proposed [6, 8], large-scale RCTs have not demonstrated consistent improvements in functional outcomes when mobilization is implemented without sufficient standardization or intensity [16].

Thus, while both high-protein nutrition and early mobilization are recognized as important ICU interventions, their effects may be limited when applied independently. Evidence from basic research suggests that muscle protein synthesis is most effectively promoted by the synergistic interaction of nutritional and mechanical stimuli [17–19]. This finding implies that the benefits of high-protein nutrition can only be realized when sufficient mobilization intensity is ensured. At the molecular level, resistance-type muscle contraction activates anabolic signaling pathways, particularly the mammalian target of rapamycin complex 1 (mTORC1), which plays a central role in regulating muscle protein synthesis. Essential amino acids, especially leucine, act as key upstream regulators of mTORC1 signaling; however, nutrient-driven stimulation of muscle protein synthesis is transient when provided alone [19]. Mechanical loading through exercise prolongs and amplifies amino acid-induced anabolic signaling, providing a biological rationale for expecting synergistic effects between high-protein intake and high-intensity mobilization.

Emerging evidence suggesting a U-shaped relationship between protein intake and outcomes should be interpreted in the context of limited or heterogeneous mechanical loading in prior trials. In most large RCTs evaluating higher protein provision, mobilization intensity was low, poorly standardized, or not systematically reported. Under such conditions, excess amino acid availability may not be effectively utilized for muscle protein synthesis and could instead contribute to metabolic burden. Therefore, we hypothesize that the optimal protein dose is not fixed, but rather depends on the presence of sufficient mechanical stimuli. In this framework, higher protein provision may be beneficial only when adequate resistance-type mobilization is ensured.

However, in real-world practice, achieving high-intensity mobilization, particularly out-of-bed activities such as standing and walking, remains challenging owing to personnel and equipment constraints [20, 21]. Such variability may obscure the clinical benefits of nutritional interventions. To address this issue, we previously reported that the use of a mobile patient lift (MPL, Golvo® 9000 lowBase (Hillrom BV, Amsterdam, The Netherlands)) enables mechanically ventilated patients to achieve early standing and improves functional independence during their ICU stay [22]. Based on these considerations, we hypothesize that higher protein intake enhances functional recovery only when combined with standardized high-intensity mobilization, whereas protein supplementation alone may be insufficient to improve functional outcomes in critically ill patients. Building on this, the present study aims to establish a realistic and standardized approach to high-intensity mobilization using an MPL for all eligible patients, and to evaluate the clinical significance of high-protein nutrition in this context through a single-center, participant- and assessor-blinded, randomized controlled trial. The primary objective of this randomized controlled trial is to evaluate whether higher protein intake improves functional recovery in ICU patients receiving enteral nutrition when combined with standardized early high-intensity mobilization.

## Methods

This protocol was developed in accordance with the SPIRIT guidelines.

### Design and settings

This prospective single-center, participant- and assessor-blinded, randomized controlled trial (RCT) will be conducted in the ICU of Toho University Omori Medical Center, a tertiary emergency care facility affiliated with Toho University in Tokyo, Japan. The ICU comprises both a general ICU and an emergency ICU managed by the institution.

The study protocol has been approved by the Ethics Committee of the Faculty of Medicine at Toho University (approval number: T2024-2411). Written informed consent will be obtained prior to enrollment. For patients who are conscious and able to make decisions, consent will be obtained directly; for those who lack decision-making capacity, consent will instead be sought from family members or legally authorized representatives.

No independent data safety monitoring board was established due to the pragmatic, low-risk nature of the trial. Participant safety will be ensured through routine ICU clinical governance, with continuous monitoring for adverse events related to nutrition therapy and mobilization. Safety data will be reviewed periodically by the principal investigator, and serious adverse events will be reported to the institutional ethics committee in accordance with local requirements. The trial may be suspended or terminated if unexpected safety concerns arise.

### Participants

Patient enrollment will begin on September 30, 2025. Eligible participants will be critically ill adults aged ≥ 18 years who are admitted to either the general ICU or emergency ICU, are independent in activities of daily living prior to admission, and are expected to require mechanical ventilation for at least 48 h. Patients receiving extracorporeal membrane oxygenation (ECMO) will also be included.

Exclusion criteria will include the following: patients admitted for scheduled surgery; those with a body weight ≥ 200 kg, exceeding the device load limit; patients admitted with neuromuscular diseases; those who undergo surgery resulting in lower-limb amputation or weight-bearing restriction after admission; patients transferred from another hospital after more than 48 h of mechanical ventilation; those unable to receive enteral nutrition; and patients with a life expectancy of < 7 days of ICU admission.

Neuromuscular diseases include conditions such as muscular dystrophies, amyotrophic lateral sclerosis, inflammatory or metabolic myopathies, Guillain-Barré syndrome, myasthenia gravis, chronic inflammatory demyelinating polyneuropathy, and other neuromuscular disorders associated with significant motor weakness. These conditions were excluded because disease-specific motor weakness, whether acute or chronic, may substantially affect baseline mobility and responsiveness to rehabilitation, thereby confounding the evaluation of functional recovery using the motor subscale of the Functional Independence Measure (FIM motor score).

### Rehabilitation protocol

In this trial, “high-intensity mobilization” is operationally defined as achieving out-of-bed mobilization at the level of standing or higher (e.g., standing or stepping) at an earlier stage of ICU stay than would be feasible with conventional mobilization methods, facilitated by the use of an MPL.

**Fig. 1:**
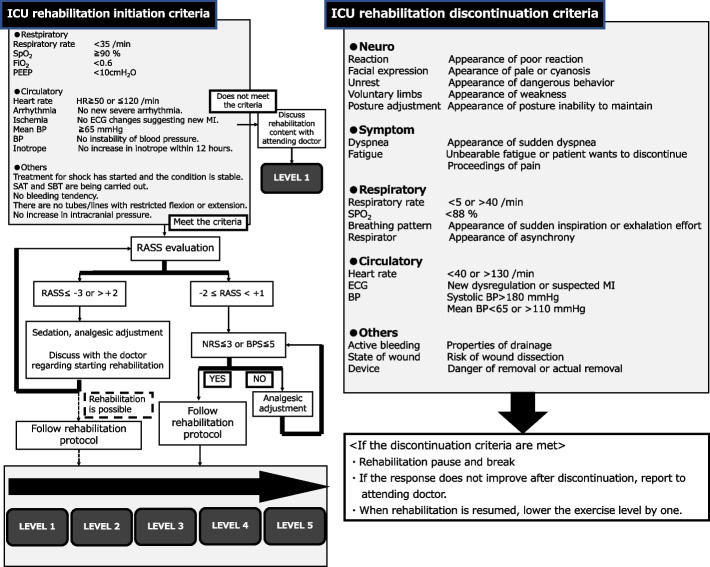
ICU rehabilitation initiation and discontinuation criteria. The flowchart summarizes the criteria for initiating and discontinuing ICU rehabilitation.The left panel describes the physiological and safety requirements for rehabilitation initiation, including respiratory, circulatory, and neurological stability, as well as sedation and analgesic evaluation (RASS, NRS, and BPS). The right panel outlines discontinuation criteria such as neurological changes, respiratory or circulatory instability, uncontrolled pain, or device-related risks. When discontinuation criteria are met, rehabilitation should be paused and restarted at a lower intensity level once the patient’s condition stabilizes. Abbreviations: MI, myocardial infarction; BP, blood pressure; SAT, spontaneous awakening trial; SBT, spontaneous breathing trial; RASS, Richmond Agitation-Sedation Scale; NRS, numerical rating scale; BPS, behavioral pain scale

**Fig. 2:**
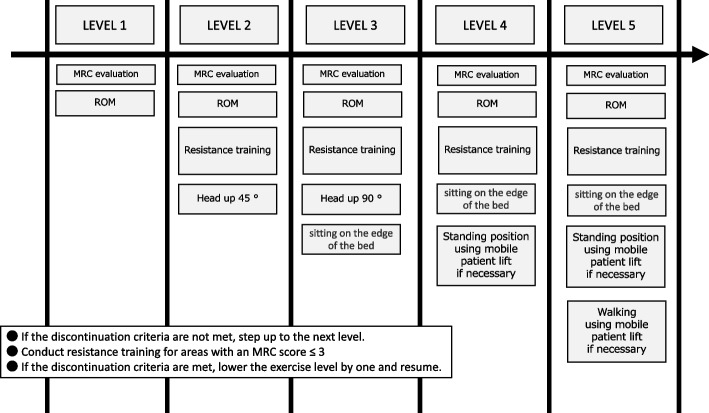
Stepwise ICU rehabilitation protocol. The rehabilitation program is divided into five progressive levels. Level 1 consists of MRC muscle strength and ROM exercises. Subsequent levels involve resistance training, heads-up positioning, sitting on the edge of the bed, and standing or walking using a mobile patient lift when necessary. Progression to the next level is permitted if the discontinuation criteria are not met; if the discontinuation criteria are met, rehabilitation is resumed at one level lower. Resistance training should be conducted for areas with an MRC score ≤ 3. Abbreviations: MRC, Medical Research Council; ROM, range of motion

The criteria for initiating and discontinuing rehabilitation will follow those established in our previous studies [22, 23]. The initial rehabilitation level will be determined through discussion among physicians, nurses, and physical therapists (Fig. 1). Rehabilitation sessions will be conducted daily and will follow a stepwise progression, with each session set at a higher intensity than the preceding session (Fig. 2). Following the initial successful achievement of sitting at the edge of the bed, patients will undergo early high-intensity rehabilitation (standing or higher) using a MPL. Our prior research demonstrated that the use of an MPL enables patients to achieve standing earlier than that achieved with standard care [22]. For patients who are capable of standing or higher-level mobilization without MPL support, the device will not be required. Thus, the purpose of this protocol is to create a cohort in which earlier and more intensive rehabilitation can be performed using the MPL. Rehabilitation with MPL will continue until patients are transferred from the ICU to the general ward.

Mobilization is delivered daily, including weekends. On weekdays, mobilization is led by physiotherapists; on weekends, mobilization at the predefined high-intensity level (standing or higher) is maintained by trained ICU nurses (and physicians when needed) using the mobile patient lift, following the same standardized protocol and safety criteria. This approach was used in our previous MPL study [22] and was feasible without a physiotherapist present on weekends. Mobilization will be performed twice daily and, when feasible, up to three times daily. The appropriate number of sessions will be determined through discussion among physicians and nurses, with adjustments made as needed.

In cases where the patient refuses mobilization, the response will depend on the underlying reason. If refusal is due to pain or fever, symptomatic treatment will be provided. If refusal is due to fatigue, the session will be rescheduled. If patients exhibit depressive symptoms and decline participation, staff will provide supportive listening and ensure that no coercion is applied. When clinically indicated, consultation with appropriate specialists (e.g., psychiatry or psychosomatic medicine) will be arranged according to standard clinical practice. However, the patient will be approached again, and if consent is obtained, mobilization will be performed.

The frequency and duration of each mobilization session will be routinely documented in the electronic medical records as part of standard ICU practice and will be extracted for analysis.

### Patient management

Daily patient management will follow the ABCDE bundle [24]. Once respiratory and circulatory stability is achieved, continuous sedation will be discontinued as early as possible, followed by a spontaneous awakening trial. Subsequently, a spontaneous breathing trial will be performed, and readiness for weaning from mechanical ventilation will be assessed daily. Delirium will be managed primarily through adequate analgesia and restoration of the circadian rhythm, than pharmacological interventions whenever possible. However, if agitation persists despite these measures, pharmacological therapy will be considered. Furthermore, as described earlier, physicians, nurses, and physical therapists will collaborate and communicate closely to plan rehabilitation, with early mobilization implemented as part of standard care.

### Nutrition protocol

In this study, the day of ICU admission will be designated as Day 0, with the following day as Day 1. Nutritional therapy is delivered primarily via enteral nutrition in all eligible patients. All patients will receive small-volume nutrition on Days 1 and 2 (10–20 mL/h). On Days 3 and 6, resting energy expenditure (REE) will be measured using indirect calorimetry. Based on these measurements, the target for total caloric intake will be set at 70% of REE from Days 3 to 5, and increased to 100% of REE from Day 6 onward [9]. Nutritional support will be delivered continuously for 21 h per day, with a 3-h interruption, until the patient begins oral intake.

Protein requirements will be calculated based on the patient’s usual body weight prior to ICU admission. In patients with obesity (body mass index ≥ 30 kg/m^2^), protein targets will be calculated using ideal body weight, defined as the body weight corresponding to a BMI of 22 kg/m^2^.

For nutrition planning, both the type and volume of formula will be automatically determined using an Excel-based tool with VBA macros, incorporating measured REE, the target protein dose, and the infusion rate of propofol at the time of planning. The tool will search for a combination in which both energy and protein intake fall within 100–110% of the targets. If no feasible combination is identified, the output will be left blank, and the final prescription will be determined at the discretion of the attending physician, who will also aim to achieve 100–110% of the targets for both energy and protein intake. This Excel-based tool was developed for clinical implementation to standardize nutritional prescriptions and does not represent a novel measurement or analytical method. Formal validation against external reference standards has not been performed; however, all calculations are based on established nutritional formulas and routinely collected clinical data.

Preparation and delivery of nutritional formulas will be performed by ICU nurses or dietitians who are not involved in outcome assessment. Participants and outcome assessors will remain blinded to group allocation throughout the study period. Given that differences in protein dose are not visually apparent at the bedside and that nutritional delivery follows standard clinical workflows, blinding is considered feasible and robust. Attending physicians are aware of the assigned protein targets for clinical safety reasons but not involved in outcome assessment.

If clinical circumstances such as persistent shock, gastrointestinal intolerance, or diarrhea necessitate modification of nutritional support, or if initiation or escalation of feeding is delayed, the attending physician may revise the plan using indirect calorimetry and the planning tool, based on the prevailing conditions. The protein source was not protocolized and followed standard clinical practice; however, the same commercially available enteral formulas were used in both groups, and no systematic differences in protein source (e.g., whey- vs. casein-predominant formulations) were intended between groups. The intervention targeted protein dose rather than protein composition. When enteral nutrition alone is insufficient to meet energy requirements, commercially available parenteral nutrition formulations containing amino acids may be used in accordance with standard ICU practice. Isolated amino acid infusion will not be used, as achieving the target protein dose via amino acid infusion would require excessive fluid volume and may increase the risk of volume overload. Dextrose-only parenteral nutrition will not be used. Adjustment of protein delivery will be primarily achieved through enteral nutrition formula selection and/or supplementation with protein powder.

Indirect calorimetry is limited in specific clinical situations, such as patients with chronic obstructive pulmonary disease, high levels of positive end-expiratory pressure, or high inspired oxygen fractions. However, it is considered the most accurate method for estimating energy expenditure in mechanically ventilated ICU patients and is therefore used whenever technically feasible. These limitations are acknowledged, and measurements will be interpreted in the context of the patient’s clinical condition. For patients receiving ECMO, REE will be estimated as 20 kcal/kg because indirect calorimetry is less reliable in this setting [9].

Renal function will be monitored daily during the intervention period using serum creatinine levels, urine output, and blood urea nitrogen.

The type and volume of enteral nutrition administered are routinely documented in the electronic medical records and daily temperature charts as part of standard ICU care. Actual daily protein intake will be calculated from these records to assess adherence to the assigned protein and calorie target. Adjustments to protein delivery may be made by the attending physician based on clinical conditions such as intolerance or renal deterioration; the actual delivered protein dose will be documented and analyzed.

Details of how nutrition delivery, mobilization, and safety parameters are routinely monitored and documented as part of standard ICU care are summarized in Table 1.

**Table 1:** Routine monitoring and documentation of nutrition delivery, mobilization, and safety parameters

Domain	What is monitored	How it is documented	Clinical oversight
Protein delivery	Daily protein intake (g/kg/day)	Electronic medical record (nutrition orders and intake records)	Adjusted by attending physician based on clinical condition
Energy delivery	Daily caloric intake (kcal/kg/day)	Electronic medical record / nutrition chart	Managed as part of routine ICU care
Mobilization	Frequency and type of mobilization sessions	Physical therapy and nursing notes in the medical record	Conducted according to patient tolerance

Protein tolerance and gastrointestinal intolerance (e.g., vomiting, diarrhea, abdominal distension, or interruption of enteral feeding) are routinely assessed and documented as part of standard ICU nursing and medical records.

### Interventions

The intervention in this study will not involve differences in the intensity or frequency of rehabilitation; the focus will be on modifying protein intake between the two groups. In the control group, nutritional support will target the lower limit of protein intake recommended in current guidelines (1.2 g/kg/day) [9, 10]. In contrast, participants in the intervention group will receive a higher protein intake of 1.8 g/kg/day. As described previously, both caloric and protein intake will be adjusted to remain within 100–110% of the prescribed targets. Although European Society for Clinical Nutrition and Metabolism guidelines suggest higher protein targets for selected populations, such as trauma or obese patients [9], the present trial is not designed to individualize protein prescription based on diagnostic subgroups, but rather to evaluate the effect of two predefined protein targets under standardized mobilization conditions. Randomization is expected to distribute such patient characteristics evenly between groups.

The core intervention period will continue from Day 3, when protocolized protein delivery is initiated, until ICU discharge, death, or the initiation of oral intake, whichever occurs first, for a maximum of 28 days from randomization. During this period, standardized high-intensity mobilization will be provided as part of routine ICU care to all eligible patients.

### Randomization

Written informed consent will be obtained from patients who are expected to require mechanical ventilation for > 48 h. Once consent is obtained, randomization will be performed. The consent process will be completed within 24 h of initiating mechanical ventilation.

Randomization will be conducted using a variable block method, with block sizes of four, six, or eight selected at random, and patients will then be allocated to the two groups in a 1:1 ratio. Allocation will be performed by an independent third party who is not involved in clinical care or data analysis. To ensure allocation concealment, clinical staff will be informed of the assigned group only after patient enrollment.

### Blinding and masking procedures

This trial will employ a participant- and assessor-blinded design, in which participants remain unaware of their group allocation. Physicians and nurses cannot be blinded owing to the nature of the intervention; however, the primary outcome, FIM motor score, will be assessed by an independent physical therapist who is blinded to group assignment, thereby ensuring assessor blinding. The assessor will be an experienced clinician routinely involved in FIM evaluation.

### Sample size estimation

Our previous study was the first to investigate early mobilization of ICU patients using an MPL [22]. However, because the primary outcome in the present study differs, no optimal prior data are available to directly inform the sample size calculation. Therefore, we referred to previous studies on post-stroke patients and adopted a standard deviation (SD) of 20 and a minimal clinically important difference of 17 for the FIM motor score [25].

As there is no established SD or MCID for the FIM motor score at hospital discharge for mechanically ventilated ICU survivors, we used estimates from the post-stroke rehabilitation literature, where FIM is most extensively validated. Variability in ICU survivors may plausibly be larger owing to heterogeneity in diagnoses and ICU-related factors (e.g., sedation, delirium, complications), which could lead to underpowering; however, the direction and magnitude of this difference are uncertain.

The sample size was calculated using Power and Sample Size Calculation software (PS, version 3.1.2, Vanderbilt University, Nashville, TN, USA). With a two-sided *α* of 0.05 and *β* of 0.95, the required sample size was 37 patients per group, yielding a total of 74. To account for an anticipated 15% dropout rate, the final sample size was set at 90 participants.

The feasibility of recruiting 90 participants over ~ 20 months is supported by prior experience from our previous single-center MPL study in the same ICU, in which 92 patients were enrolled within approximately 20 months [22].

### Data collection

Baseline characteristics will be collected at the start of the intervention, and will include demographic and clinical data: age, sex, body mass index, primary diagnosis, and comorbidities assessed using the Charlson Comorbidity Index [26]. Severity of illness will be measured using the Acute Physiology and Chronic Health Evaluation (APACHE) II score. Additional clinical variables will include the presence of acute kidney injury (AKI) according to the Kidney Disease: Improving Global Outcomes criteria [27], presence of diabetes, score on the Malnutrition Universal Screening Tool [28], and the presence of malnutrition defined by the Global Leadership Initiative on Malnutrition criteria [29]. Furthermore, body composition will be assessed at enrollment using a bioelectrical impedance analyzer (InBody BWA2.0S; InBody Japan, Tokyo, Japan) to determine parameters including skeletal muscle mass, skeletal muscle index, fat-free mass index, and phase angle. Baseline FIM scores will not be collected because most patients are unable to undergo functional assessment at ICU admission due to sedation, mechanical ventilation, or impaired consciousness. Therefore, FIM was assessed only at hospital discharge, consistent with the study objective of evaluating post-ICU functional recovery.

Nutritional data will include the number of days from admission to initiation of enteral nutrition, the number of days until the target protein dose was achieved, and the mean intake of caloric, protein, parenteral caloric, enteral caloric, parenteral amino acid, and enteral protein during the intervention period. These data will be analyzed separately for Days 0–1 and from Day 3 onward.

Rehabilitation-related data will include the number of days from admission to initiation of mobilization, number of days mobilization was performed, and the ICU Mobility Scale score at ICU discharge [30].

In addition, treatment-related data will include the use of renal replacement therapy, use of ECMO, and the duration of mechanical ventilation.

### Outcome

The primary outcome will be the total motor domain score of FIM at hospital discharge. The FIM motor score at hospital discharge was selected as the primary outcome because it represents the earliest clinically stable time point at which the cumulative effects of ICU-based interventions can be meaningfully assessed. FIM at ICU discharge may be influenced by non-clinical factors such as bed availability and transfer policies, and may underestimate functional recovery due to ongoing critical illness and sedation effects. In contrast, longer-term follow-up after discharge is more susceptible to loss to follow-up and confounding by post-ICU rehabilitation and social factors. Therefore, assessment at hospital discharge was considered the most appropriate balance between clinical relevance, feasibility, and internal validity.

Secondary outcomes and their assessment time points will be as follows:

- At ICU discharge: Functional Status Score for the ICU [31], Medical Research Council (MRC) score, and ICU length of stay.
- At 14 days after ICU discharge: FIM motor, cognitive subscale of FIM (FIM cog), Mini-Mental State Examination (MMSE), body composition parameters, MRC score, and Brief Pain Inventory (BPI).
- At hospital discharge: MRC score, FIM cog, body composition parameters, BPI, length of hospital stay, and mortality.
- At 60 days after hospital discharge: FIM motor score, MRC score, FIM cog, MMSE score, body composition parameters, and BPI.

Follow-up at 60 days post-discharge will be conducted during an outpatient visit scheduled at the time of discharge. For patients who die or cannot be assessed owing to severe consciousness disturbance, the FIM motor and FIM cog will be recorded at their minimum values (13 and 5, respectively), the MRC score will be assigned as 0, and the BPI will be recorded as missing. For patients who die before hospital discharge, FIM scores will be assigned as the minimum possible values (motor score 13 and cognitive score 5). This approach reflects that death represents the worst possible functional outcome and ensures that mortality is appropriately incorporated into the analysis of functional recovery rather than treated as missing data.

**Fig. 3:**
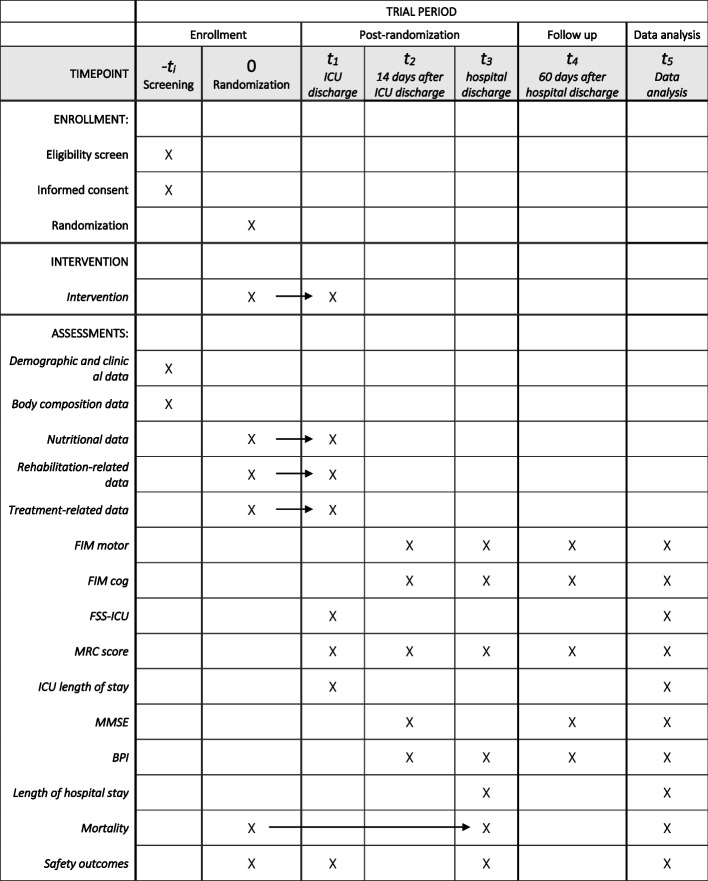
SPIRIT figure: Schedule of enrolment, interventions, and assessments. The schedule of enrolment, interventions, and assessments follows the SPIRIT 2025 guidelines. Abbreviations: ICU, intensive care unit; FIM, Functional Independence Measure; FSS-ICU, Functional Status Score for the ICU; MRC, Medical Research Council; MMSE, Mini-Mental State Examination; BPI, Brief Pain Inventory

Safety outcomes will include serum creatinine level at hospital discharge, maximum blood urea nitrogen level during the intervention, mean insulin use, and maximum blood glucose levels. In addition, mobilization-related safety outcomes will include accidental removal of tubes or devices, the number of mobilization sessions discontinued, and reasons for discontinuation (respiratory, circulatory, neurological, fatigue, or other causes). The schedule of enrolment, interventions, and assessments follows the SPIRIT 2025 guidelines (Fig. 3).

### Statistical analyses

After data collection is complete, continuous variables will be presented as mean ± SD or as median with interquartile range (IQR), and categorical variables as numbers with percentages. Baseline characteristics and study outcomes will be compared between groups using the Student’s t-test or Mann–Whitney *U* test for continuous variables, and the chi-square test or Fisher’s exact test for categorical variables. All outcome analyses will be conducted according to the intention-to-treat principle.

The primary analysis will compare outcomes between groups according to the intention-to-treat principle.

As a sensitivity analysis, analysis of covariance (ANCOVA) will be performed to explore the robustness of the primary findings to variability in intervention implementation. As the actual duration of high-protein nutrition and high-intensity mobilization may vary among participants despite randomization, these variables will be included as covariates to account for differences in intervention exposure rather than to adjust the primary treatment effect.

Covariates included in the ANCOVA will be pre-specified based on clinical relevance and trial design considerations, not selected in a data-driven manner. Importantly, these analyses are intended as exploratory sensitivity analyses to aid interpretation of the results and will not replace the primary intention-to-treat comparisons.

Furthermore, sensitivity analyses will be performed with adjustment for the presence of acute kidney injury and use of renal replacement therapy (RRT).

For all analyses, missing data will be addressed under the assumption of missing at random using multiple imputation. Given the possibility of non-random loss to follow-up after hospital discharge, sensitivity analyses exploring departures from the missing-at-random assumption (e.g., pattern-mixture or delta-adjustment approaches) will be conducted, and the extent and patterns of missingness will be reported. Variables related to missingness or outcomes—including group allocation, age, sex, Glasgow Coma Scale, ICU length of stay, and APACHE II score—will be incorporated in the imputation model. The specification of the imputation model will be informed by assessment of missing data patterns and inter-variable associations to ensure model adequacy. This approach will also be applied to post-discharge follow-up outcomes, including those assessed at 60 days, to account for missing data due to transfer or loss of contact.

Model assumptions for ANCOVA will be evaluated using standard diagnostic procedures, including inspection of residual distributions and assessment of homoscedasticity. If major violations are identified, appropriate transformations or robust alternatives will be considered and reported.

No interim analyses will be conducted. All statistical analyses will be performed using R (version 4.5.0), and all statistical tests will be two-sided. Statistical significance will be set at 0.05 across all analyses.

### Subgroup analysis

Subgroup analyses will be performed according to sex, presence or absence of AKI, use of RRT, presence or absence of diabetes, duration of the intervention period, duration of mobilization, and baseline severity based on the APACHE II score.

## Discussion

This study is designed to evaluate whether high-protein nutrition contributes to improved physical function in critically ill patients, specifically focusing on a cohort undergoing earlier and more intensive mobilization than is typical in conventional practice. The findings are expected to provide valuable evidence on the potential synergistic effects of high-protein nutrition and rehabilitation.

Previous studies have shown that both high-protein nutrition and early mobilization, when implemented separately, offer only modest benefits. Moreover, several trials have investigated the combined use of nutrition and exercise therapy. For example, RCTs conducted by Wu et al. [32] and de Azevedo et al. [33] examined the effect of combined interventions. In the study by Wu et al. [32], a four-arm comparison was performed to assess resistance training with or without beta-hydroxy-beta-methylbutyrate (HMB) supplementation. Although the intervention improved certain aspects of physical function, the nutritional component was based on HMB rather than dietary protein intake set at a level that could be classified as high-protein nutrition.

In the study by de Azevedo et al. [33], high-protein nutrition (2.0–2.2 g/kg/day) was combined with exercise using a cycle ergometer. While the protein intake satisfied the criteria for high-protein nutrition, the simultaneous intensification of both exercise and diet made it difficult to isolate the independent contribution of each intervention. Moreover, the cycle ergometer primarily provides aerobic exercise, raising concerns regarding variability in exercise intensity and limited muscle loading.

In contrast, the present study will employ an MPL to deliver standardized, high-intensity mobilization (defined as standing or higher) for all participants, thereby ensuring consistency in exercise intensity and implementation. By using protein dose as the sole intervention variable, this study will enable a more rigorous evaluation of the direct effects of protein intake on physical function. Furthermore, MPL can be safely applied even in patients with mild consciousness disturbances or muscle weakness, enhancing both the standardization of rehabilitation and the inclusiveness of the intervention cohort.

This study is designed as a participant- and assessor-blinded randomized controlled trial, in which patients will remain blinded, while outcome assessors will also be blinded to group allocation to ensure reliability of the results. Another notable feature is the use of individualized caloric prescriptions based on REE measured through indirect calorimetry. This approach minimizes the risk of metabolic complications associated with overfeeding while ensuring appropriate nutritional management. Moreover, by balancing caloric targets with protein goals and employing an automated VBA-based calculation tool to determine formula and supplemental products, the study will enhance both precision and reproducibility in nutrition planning.

Several potential limitations should also be acknowledged. First, this is a single-center study, which may limit the generalizability of the findings. The single-site design was chosen for practical reasons, as relatively few ICUs are currently equipped with MPL, and its use requires specialized training. Second, the participant- and assessor-blinded design cannot completely eliminate the risk of treatment bias. To mitigate this concern, assessor blinding has been incorporated to minimize potential bias in outcome evaluation. Third, the duration of the intervention may vary among patients, with longer exposure potentially leading to greater functional improvement. To address this, ANCOVA including intervention duration as a covariate will be performed, and planned subgroup analyses will further account for potential heterogeneity.

Although recent post-hoc analyses have suggested potential harm of higher protein intake in patients with severe AKI [13], current guidelines do not recommend routine protein restriction.

Our protocol incorporates daily renal monitoring, predefined safety-driven adjustments, and planned subgroup analyses to address this concern.

## Trial status

Protocol version 1.0 was finalized on September 10, 2025, following approval by the institutional ethics committee. The trial was registered on September 29, 2025. Patient recruitment began on September 30, 2025, and is expected to be completed by May 31, 2027. At the time of manuscript submission, recruitment had begun, but no participants had yet been enrolled.

## Supplementary Information

Supplementary Material 1. SPIRIT 2025 checklistR3.

## Data Availability

The datasets generated and/or analyzed during the current study are not publicly available due to patient confidentiality but are available from the corresponding author on reasonable request.

## Abbreviations

AKI: Acute kidney injury
APACHE II: Acute Physiology and Chronic Health Evaluation II
BPI: Brief Pain Inventory
ECMO: Extracorporeal membrane oxygenation
FIM: Functional Independence Measure
ICU: Intensive care unit
MMSE: Mini-Mental State Examination
MPL: Mobile patient lift
REE: Resting energy expenditure
