# Correction: High-protein enteral nutrition in intensive care unit patients undergoing advanced early mobilization: protocol for a randomized controlled trial

**DOI:** 10.1186/s13063-026-09898-8

**Published:** 2026-07-09

**Authors:** Ginga Suzuki, Yuji Iwanami, Masayoshi Nakazawa, Shunta Yoshioka, Masashi Furuta, Masaru Nakanishi, Hiromi Kanayama, Kohei Ishikawa, Saria Nishioka, Toshimitsu Kobori, Yuka Masuyama, Saki Yamamoto, Yui Shimanuki, Hibiki Serizawa, Yoshimi Nakamichi, Mitsuru Honda, Naohiro Washizawa, Ikuko Okuni

**Affiliations:** 1 Critical Care Center, Toho University Omori Medical Center, 6-11-1, Omori Nish, Ota-ku, Tokyo, Japan; 2 Department of Rehabilitation Medicine, Toho University School of Medicine, 6-11-1, Omori Nishi, Ota-ku, Tokyo, Japan; 3 Department of Nutrition Management, Toho University Omori Medical Center, 6-11-1, Omori Nishi, Ota-ku, Tokyo, Japan

**Correction: Trials 27, 247 (2026)**

**https://doi.org/10.1186/s13063-026-09584-9**

Following the publication of the original article [1], we were notified of an error in the Methods section of the Abstract.

The relevant sentence originally read:

“This single-center, prospective, participant- and assessor-blinded randomized controlled trial with a planned sample size of 92 critically ill patients will be conducted in the ICU of a tertiary emergency care facility.”

The corrected sentence should read:

“This single-center, prospective, participant- and assessor-blinded randomized controlled trial with a planned sample size of 90 critically ill patients will be conducted in the ICU of a tertiary emergency care facility.”

This error is limited to the sample size stated in the Abstract. The sample size calculation, study design, statistical analysis plan, and conclusions of the protocol remain unchanged.

The original article has been corrected.
